# The GATA Factor *elt-1* Regulates *C*. *elegans* Developmental Timing by Promoting Expression of the *let-7* Family MicroRNAs

**DOI:** 10.1371/journal.pgen.1005099

**Published:** 2015-03-27

**Authors:** Max L. Cohen, Sunhong Kim, Kiyokazu Morita, Seong Heon Kim, Min Han

**Affiliations:** 1 Howard Hughes Medical Institute and Department of Molecular, Cellular, and Developmental Biology, University of Colorado, Boulder, Colorado, United States of America; 2 Incurable Disease Therapeutics Research Center, Korea Research Institute of Bioscience and Biotechnology, Cheongju, Republic of Korea; 3 Department of Biomolecular Science, University of Science and Technology, Daejeon, Republic of Korea; Harvard University, UNITED STATES

## Abstract

Postembryonic development in *Caenorhabditis elegans* is a powerful model for the study of the temporal regulation of development and for the roles of microRNAs in controlling gene expression. Stable switch-like changes in gene expression occur during development as stage-specific microRNAs are expressed and subsequently down-regulate other stage-specific factors, driving developmental progression. Key genes in this regulatory network are phylogenetically conserved and include the post-transcriptional microRNA repressor LIN-28; the nuclear hormone receptor DAF-12; and the microRNAs LIN-4, LET-7, and the three LET-7 family miRNAs (miR-48, miR-84, and miR-241). DAF-12 is known to regulate transcription of miR-48, miR-84 and miR-241, but its contribution is insufficient to account for all of the transcriptional regulation implied by the mutant phenotypes. In this work, the GATA-family transcription factor ELT-1 is identified from a genetic enhancer screen as a regulator of developmental timing in parallel to DAF-12, and is shown to do so by promoting the expression of the LET-7, miR-48, miR-84, and miR-241 microRNAs. The role of ELT-1 in developmental timing is shown to be separate from its role in cell-fate maintenance during post-embryonic development. In addition, analysis of Chromatin Immnoprecipitation (ChIP) data from the modENCODE project and this work suggest that the contribution of ELT-1 to the control of *let-7* family microRNA expression is likely through direct transcription regulation.

## Author Summary

In the nematode roundworm *C*. *elegans*, seam cells, a type of adult stem cell, divide in a completely predictable manner throughout post-embryonic development. Study of the control of the timing of these cells’ division and differentiation led to the discovery of the first microRNAs, which are small non-coding RNAs that regulate the expression of protein-coding mRNAs, but knowledge of the regulation of expression of microRNAs themselves within *C*. *elegans* stem cells remains incomplete. In this study, the GATA-family transcription factor *elt-1*, known to be important for the formation and maintenance of tissues during embryonic and post-embryonic development, is found to regulate the expression of *let-7* family microRNAs in stem cells during late developmental stages. It is found to do so redundantly with *daf-12*, the only other transcription factor previously known to directly regulate microRNA expression in *C*. *elegans*. In addition, the presence of ELT-1 *in vivo* binding near microRNA coding DNA sequences suggests that its contribution to the regulation of microRNA expression is likely through direct regulation of transcription. Stem cells are important in development, tissue homeostasis, and malignancy, so additional knowledge of the mechanisms underlying their maintenance, renewal, and differentiation is of broad interest.

## Introduction

Extensive study of postembryonic development in the nematode *Caenorhabditis elegans* has advanced our understanding of the temporal regulation of development and the roles of microRNAs (miRNAs) in controlling gene expression [1–5]. In *C*. *elegans*, developmental timing is regulated by the heterochronic gene network, which directs the transitions among discrete developmental stages largely by initiating the stage-specific of miRNAs that down-regulate other stage-specific factors [6–8]. Many gene products of the *C*. *elegans* heterochronic regulatory network are conserved in metazoans, including the LET-7 family of miRNAs and LIN-28, a post-transcriptional repressor of these miRNAs [9–11]. LET-7 family miRNAs regulate the expression of multiple targets, including LIN-41, and the LIN-28-LET-7-LIN-41 pathway has been shown to regulate differentiated states of stem cells in both *C*. *elegans* and mammals [3,4,12–17]. The LIN-28-LET-7 axis is important in human physiology and disease, as it is involved in induced pluripotency [17–19], adult intestinal stem cell function [20], tissue repair [21], and malignancy [22,23].

During normal development, dafachronic acid steroid hormones are synthesized by *C*. *elegans* in response to favorable growth conditions [24]. They stimulate the nuclear hormone receptor (NHR) DAF-12, the vitamin D NHR ortholog, to promote progression from the 2^nd^ larval stage (L2) to the 3^rd^ larval stage (L3) [24–26] by, in part, initiating expression of the LET*-*7 family of miRNAs, miR-48, miR-84, and miR-241, during or near the L2-to-L3 molt [27,28]. In this way, the nuclear-hormone receptor DAF-12 acts as a key switch in the regulation of developmental fate [27–29]. Expression of miRs has been proposed to drive transition from one larval stage to the next [8]. DAF-12/NHR is known to regulate miRNA expression in this system [7], but cannot itself account for all of the upstream transcriptional regulation of the LET-7 family of miRNA, as its *null* phenotype is much weaker than that of the LET-7 family itself [25,26,30]. LET-7 is known to be under-expressed in both *daf-12* mutants [28] and *alg-1* mutants [31], and a portion of its promoter region has been identified to be required for correct temporal expression [32], but the factor(s) that directly regulate its transcription are not yet known. Additionally, the transcriptional regulation of LIN-4 remains largely unknown. A previous study determined that LIN-66 provides regulation of developmental timing in parallel to *daf-12*, but the molecular function of the LIN-66 protein remains unknown [33]. Recently, the PERIOD homolog *lin-42* has been found to negatively regulate the expression of multiple microRNAs, including LIN-4 and LET-7 [34]. The presence of other regulatory factors that act on the transcription of these miRs is implied, and the identification of these factors would significantly advance our understanding of developmental timing regulation as well of miRNA function in general.

In this study, we performed a forward genetic screen to identify enhancers of the heterochronic phenotype of *daf-12(null)* animals; the purpose was to identify new factors that act in parallel to it in the regulation of the heterochronic genetic network. A partial loss-of-function allele of the GATA transcription factor *elt-1* was positionally cloned, and the role of ELT-1 in the heterochronic gene network is described.

## Results

### ELT-1 and DAF-12 redundantly regulate seam cell fate during post-embryonic development

**Fig 1:**
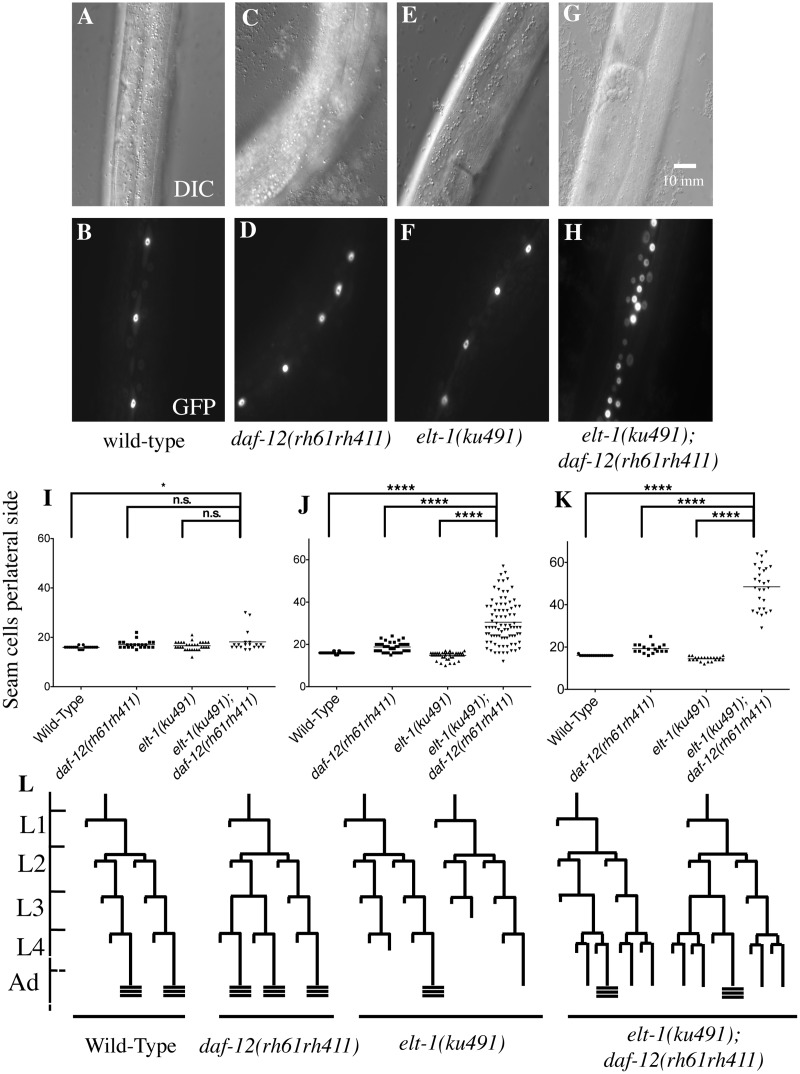
ELT-1/GATA acts in parallel to DAF-12/NHR to regulate developmental timing in *C*. *elegans*. A-H, DIC and fluorescence images of representative young-adult stage animals with the indicated genotypes, showing that seam cell numbers, visualized with a *scm*::*GFP* marker, are drastically increased in *elt-1(ku491); daf-12(rh61rh411)* double mutants but not in each single mutant. I-K, Scatter plots showing the distribution of the number of seam cells per lateral side for each genotype at L4 and young adult stages; horizontal bar is mean. L, cellular lineage diagrams for mutants, showing variable cell fate defects in the *elt-1(ku491)* mutants during the L3 and L4 stages. **** indicates p-value < 0.0001. Data for L1 to L3 stages and summary statistics of data for all developmental stages are shown in S1 Fig.

**Fig 2:**
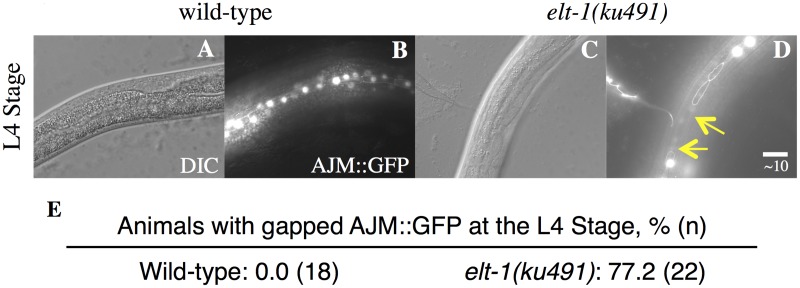
*elt-1/(ku491)* mutant animals have defective adult alae formation. A-D, DIC and GFP fluorescence images showing adherins junctions of L4 animals with indicated genotypes. Arrow in D marks the gap of AJM::GFP fluorescence. E, Percentage of L4 animals with AJM::GFP fluorescence gaps in animals with the indicated genotypes.

An EMS-mutagenesis screen was performed to identify mutations that enhance the heterochronic phenotype of *daf-12(rh61rh411)* animals. One such enhancer allele was identified and mapped to the *elt-1/GATA* gene by genetic mapping techniques including genetic and SNP markers, whole-genome shotgun sequencing to identify candidate variations, and transgene-mediated phenotype complementation. As shown in Figs. 1, 2A–2F, and S1, and Table 1, the *elt-1/GATA(ku491)* mutation causes delayed heterochronic phenotypes when animals are double-mutant for *daf-12(rh61rh411)*, but not when animals are *daf-12(+)*. Specifically, the number of seam cells in the lateral hypodermis is dramatically higher than normal during the 4^th^ larval (L4) and young adult (Y.A.) stages (Fig. 1). In addition, the double-mutants have an L4-stage bursting vulva phenotype (Table 1), similar to that caused by mutation of delayed heterochronic genes, including *let-*7 and the three other *let-7* family miR genes (*mir-48*, *mir-84*, and *mir-241*) [3,30]. These results indicate that *elt-1/GATA* has an important role in regulating developmental timing in parallel to *daf-12*. *daf-12* is known to regulate developmental timing by promoting the expression of the three LET-7 family miRNAs [27,28], suggesting that *elt-1* may regulate developmental timing by acting either in parallel to or upstream of the miRNAs.

**Table 1:** *elt-1* mutants have an L4-stage bursting vulva and defective alae formation and *elt-1(ku491)* is a partial loss-of-function allele.

Strain	RNAi	L4 Bursting Vulva		L4 Molt Alae				Young Adult Seam Cells		
		%	n	Absent (%)	Gapped (%)	Present (%)	n	SCM	Std Dev	n
wild-type	Empty vector^a^	0.0	245	0.0	0.0	100.0	27	16.0	0.4	27
*daf-12(rh61rh411)*	Empty vector^a^	0.0	183	0.0	0.0	100.0	24	18.1	1.6	24
*elt-1(ku491)*	Empty vector^a^	3.6	169	88.4	3.8	7.6	26	12.9	2.2	15
*elt-1(ku491);daf-12(rh61rh411)*	Empty vector^a^	55.1	198	88.8	0.0	11.1	18	39.7	9.8	29
*wild-type*	*elt-1*	48.7	117	100.0	0.0	0.0	21	0.5	0.8	21
*daf-12(rh61rh411)*	*elt-1*	56.7	90	90.0	5.0	5.0	20	0.2	0.4	20
*elt-1(ku491)*	*elt-1*	76.3	114	100.0	0.0	0.0	27	1.3	1.2	27
*elt-1(ku491);daf-12(rh61rh411)*	*elt-1*	94.8	231	96.4	3.6	0.0	28	2.0	2.2	28
***elt-1*genotype** ^b^	***daf-12*genotype**									
*ku491* *+*	*Wild-type*	0.0	60	0.0	0.0	100.0	19	15.9	0.3	18
*ku491* *+*	*rh61rh411*	0.0	57	0.0	0.0	100.0	18	18.3	1.8	18
*ok1002* *+*	*Wild-type*	0.0	92	0.0	0.0	100.0	18	16.0	0.3	18
*ok1002* *+*	*rh61rh411*	0.0	78	0.0	0.0	100.0	17	18.2	1.9	17
*ku491* *ok1002*	*Wild-type*	1.9	52	84.0	16.0	0.0	25	9.4^c^	2.0	25
*ku491* *ok1002*	*rh61rh411*	65.9	41	100.0	0.0	0.0	20	18.2^d^	6.6	20

^a^Phenotypes on empty-vector control RNAi were similar to the standard *E*. *coli* strain OP50.

^b^An allele of *elt-1(ku491)* linked to mutations in *unc-24* and *dpy-20* was used for these strains.

^c^For *elt-1(ku491)* animals vs *elt-1(ku491)-over-elt-1(null)* animals at the young adult stage, the p-value for the comparison of seam-cell numbers is 0.0340

^d^For *elt-1(ku491);daf-12(rh61rh411)* animals vs *elt-1(ku491)-over-elt-1(null);daf-12(rh61rh411)* animals at the young adult stage, the p-value for the comparison of seam-cell numbers is < 0.0001.

In addition to the heterochronic phenotypes that are only present when *daf-12* is null, *elt-1/GATA(ku491)* animals have *daf-12(null)*-independent defects in adult alae formation at the L4 molt and in the maintenance of seam cells during post-embryonic development (Table 1). These data are consistent with previous studies that have used post-embryonic RNAi against *elt-1/GATA* to show that it is required for the maintenance of seam-cell cell identities during post-embryonic development and for the formation of adult alae at the L4 molt [35–37]. The timing of premature differentiation of seam-cells in *elt-1(ku491)* single mutants and of excessive seam-cell divisions in the *elt-1(ku491);daf-12(rh61rh411)* double-mutants is variable but primarily during the L4 stage (Fig. 1L). Additionally, no supernumerary molts were observed, and male *elt-1/GATA(ku491)* single-mutant animals are able to cross-fertilize hermaphrodites. Among seam-cell nuclei that formed after seam-cell fusion into the hypodermal syncytium, we did not determine the proportion due to duplicate nuclei within fused cells versus in cells with completed divisions. Proliferation of seam cells and the L4 bursting vulva phenotype in *elt-1(ku491); daf-12(null)* double-mutant animals are novel phenotypes for *elt-1/GATA* and indicate that it is a heterochronic gene.

### The *elt-1/GATA(ku491)* mutant allele is a partial loss-of-function allele

As shown in Table 1, the *elt-1(ku491)* mutation is fully recessive. Animals with the *elt-1(ku491)* mutation in *trans* to a null allele, *elt-1(ok1002)*, have an equivalent or stronger phenotype for alae formation defects and L4 burst vulva when compared to *elt-1/GATA(ku491)* homozgotes. These data indicate that the *elt-1(ku491)* mutation is likely a partial loss-of-function mutation that compromises the role of the ELT-1 protein in post-embryonic developmental timing and adult alae formation, while leaving it competent to function in the embryonic specification of the hypodermis. Interestingly, there is reduced seam-cell proliferation in *elt-1(ku491)-over-elt-1(null); daf-12(rh61rh411)* animals compared to *elt-1(ku491); daf-12(rh61rh411)* animals. This is likely due to allelic haploinsufficiency of *elt-1(ku491)* for the maintenance of post-embryonic seam cell fate as, when *daf-12* is wild-type, *elt-1(ku491)-over-elt-1(null)* animals also have a decrease in the numbers of seam-cells compared to *elt-1(ku491)* single-mutants animals, and *elt-1* has previously been shown to be required for the post-embryonic maintenance of seam-cell cell fate [35–37].

The *elt-1/GATA(ku491)* mutant allele contains a C-to-T substitution in the 48^th^ base-pair of exon 5, causing a proline-to-serine missense mutation at amino-acid residue 298 of the ELT-1 protein isoform A. ELT-1 has previously been shown to contain two conserved Zinc-finger DNA binding domains, each of which contains a single C-X_2_-C-X_17_-C-X_2_-C motif [38]; proline^298^ is 6 amino acid residues C-terminal to the second cysteine of the N-terminal Zn-finger DNA binding domain. This proline specifically, and the N-terminal Zinc-finger DNA binding domain overall, is conserved among worms, fish, mice, and humans (S2 Fig). Examining the structure of the murine GATA1 Zn-finger DNA-binding domains (Protein Databank accession number 3VD6) [39], we found that the amino acid residue conserved with *C*. *elegans* ELT-1 proline^298^ is located within a hairpin fold that bring the four cysteine residues of the N-terminal Zn-finger domain near to the required Zinc molecule (S3 Fig). This suggests that the *elt-1(ku491)* mutation could potentially alter the secondary structure of the N-terminal Zn-finger domain in the ELT-1 protein by interfering with the folding required for Zinc binding, causing its functional inactivation. This presumably does not have an effect on the ability of the C-terminal domain to recognize its target sequences as the *elt-1(ku491)* mutant has a non-*null* phenotype.

Five alleles of *elt-1* were obtained from the million mutation project and examined in the presence of the *daf-12(rh61rh411)* mutation (S1 Table); two showed very mild increases in the number of seam-cells when compared to *daf-12(rh61rh411)* animals, but none showed an L4 bursting vulva phenotype or defective alae formation. These alleles contain mutations with mild effects on the ELT-1 protein, as listed in S1 Table, and have no previously described phenotype, so they likely are minor mutations that do not substantially interfere with the normal function of ELT-1, unlike the proline^298^-to-serine mutation present in *elt-1(ku491)*.

In sum, the *elt-1/GATA(ku491)* allele significantly reduces the normal function of ELT-1/GATA during post-embryonic development, likely by disrupting the DNA binding ability of its N-terminal Zn-finger domain.

### *elt-1/GATA* likely acts upstream of key heterochronic genes to control developmental timing

The well-described heterochronic gene network controls developmental timing in *C*. *elegans* [8]. To assess a possible genetic relationship between *elt-1/GATA* and key genes in the heterochronic gene network, the phenotype of *elt-1(ku491); daf-12(rh61rh411)* double-mutant animals was examined while the expression of several key heterochronic genes were each reduced by feeding RNAi, applied starting at the L1 stage. The results of this interaction analysis (Table 2) shows that the heterochronic phenotypes of *elt-1/GATA(ku491); daf-12(rh61rh411)* double-mutant animals requires normal activity of the products of the heterochronic genes *lin-14*, *lin-28*, *hbl-1*, *lin-41*, *lin-42*, and *mab-10*, as RNAi of these genes significantly reduced both the high seam cell number and busting vulva phenotypes. Additionally, these phenotypes were enhanced by knockdown of the *lin-46* gene, and the seam-cell proliferation phenotype was not affected by knockdown of the *lin-29* gene. Knockdown of *ceh-16*, which is involved in the regulation of seam-cell fate during post-embryonic development [40,41], suppressed both the high seam cell number and busting-vulva phenotypes, while RNAi of either *kin-20* or *dre-1*, which are both involved in the promotion of late larval fates [42,43], each suppressed the seam-cell proliferation phenotype but not the bursting-vulva phenotype.

**Table 2:** Epistasis analysis of *elt-1(ku491); daf-12(rh61rh411)* mutant phenotypes with RNAi of other heterochronic genes.

Strain^a^	RNAi	L4 Bursting Vulva^b^		L3 Molt Alae				L4 Molt Alae				Young Adult Stage Seam Cells^c^		
		%	n	Abs(%)	Gap(%)	Pres (%)	n	Abs (%)	Gap (%)	Pres(%)	n	#	Std Dev	n
wild-type	e.v.	0.0	245	100	0	0	17	0	0	100	42	16.0	0.4	27
*daf-12(lf)*	e.v.	0.0	183	100	0	0	12	0	0	100	34	18.1	1.6	24
*elt-1(rf)*	e.v.	3.6	169	100	0	0	12	84	16	0	50	12.9	2.2	15
*elt-1(rf); daf-12(lf)*	e.v.	55.1	198	-	-	-		95	5	0	39	39.7	9.8	29
wild-type	*lin-28*	0.0	111	0	10	90	21	0	0	100	19	12.1	1.4	20
*daf-12(lf)*	*lin-28*	0.0	197	11	6	83	18	0	10	90	20	12.0	1.7	21
*elt-1(rf)*	*lin-28*	0.4	276	-	-	-	-	97	3	0	31	11.3	1.6	28
*elt-1(rf); daf-12(lf)*	*lin-28*	15.8	76	-	-	-	-	92	6	3	36	13.3	2.4	31
wild-type	*hbl-1*	1.1	94	0	6	94	17	0	0	100	12	13.6	2.0	14
*daf-12(lf)*	*hbl-1*	2.2	89	36	43	21	14	0	0	100	10	13.4	1.4	14
*elt-1(rf)*	*hbl-1*	8.5	82	-	-	-	-	87	13	0	23	12.2	1.8	17
*elt-1(rf); daf-12(lf)*	*hbl-1*	7.1	56	-	-	-	-	85	15	0	21	12.7	3.2	19
wild-type	*lin-14*	0.0	63	36	0	64	22	8	0	92	13	14.8	0.8	13
*daf-12(lf)*	*lin-14*	0.0	39	38	0	63	16	6	0	94	15	18.6	3.5	15
*elt-1(rf)*	*lin-14*	2.9	70	-	-	-	-	95	0	5	20	11.5	1.5	21
*elt-1(rf); daf-12(lf)*	*lin-14*	22.2	63	-	-	-	-	89	5	5	19	17.4	3.2	19
wild-type	*lin-41*	0.0	50	80	20	0	15	0	0	100	13	15.5	0.6	15
*daf-12(lf)*	*lin-41*	1.6	128	56	6	39	18	7	0	93	15	21.0	2.8	16
*elt-1(rf)*	*lin-41*	15.3	59	-	-	-	-	88	12	0	17	14.1	2.0	17
*elt-1(rf); daf-12(lf)*	*lin-41*	4.1	49	-	-	-	-	94	0	6	16	21.3	3.3	16
wild-type	*lin-46*	0.0	76	100	0	0	14	0	0	100	15	16.3	0.6	15
*daf-12(lf)*	*lin-46*	0.0	46	100	0	0	17	75	0	25	16	23.2	2.7	16
*elt-1(rf)*	*lin-46*	0.0	62	-	-	-	-	100	0	0	14	13.0	1.7	14
*elt-1(rf); daf-12(lf)*	*lin-46*	78.9	57	-	-	-	-	94	6	0	17	63.8	12.4	18
wild-type	*lin-29*	-	-	-	-	-	-	93	7	0	15	16.0	0.4	15
*daf-12(lf)*	*lin-29*	-	-	-	-	-	-	100	0	0	19	19.3	2.6	19
*elt-1(rf)*	*lin-29*	-	-	-	-	-	-	100	0	0	16	13.8	1.9	16
*elt-1(rf); daf-12(lf)*	*lin-29*	-	-	-	-	-	-	100	0	0	26	41.7	8.7	27
wild-type	*lin-42*	-	-	-	-	-	-	-	-	-	-	16.1	0.78	17
*daf-12(lf)*	*lin-42*	-	-	-	-	-	-	-	-	-	-	16.6	1.08	14
*elt-1(rf)*	*lin-42*	-	-	-	-	-	-	-	-	-	-	14.3	1.54	15
*elt-1(rf); daf-12(lf)*	*lin-42*	14.4	160	-	-	-	-	-	-	-	-	23.3	5.80	20
wild-type	*kin-20*	-	-	-	-	-	-	-	-	-	-	16.1	0.5	26
*daf-12(lf)*	*kin-20*	-	-	-	-	-	-	-	-	-	-	19.8	2.2	27
*elt-1(rf)*	*kin-20*	-	-	-	-	-	-	-	-	-	-	12.8	1.7	28
*elt-1(rf); daf-12(lf)*	*kin-20*	39.4	66	-	-	-	-	-	-	-	-	23.7	4.5	37
wild-type	*mab-10*	-	-	-	-	-	-	-	-	-	-	16.7	0.6	29
*daf-12(lf)*	*mab-10*	-	-	-	-	-	-	-	-	-	-	22.2	2.2	31
*elt-1(rf)*	*mab-10*	-	-	-	-	-	-	-	-	-	-	13.6	1.7	38
*elt-1(rf); daf-12(lf)*	*mab-10*	10.5	228	-	-	-	-	-	-	-	-	30.4	6.0	38
wild-type	*ceh-16*	-	-	-	-	-	-	-	-	-	-	13.5	1.4	16
*daf-12(lf)*	*ceh-16*	-	-	-	-	-	-	-	-	-	-	16.6	1.5	16
*elt-1(rf)*	*ceh-16*	-	-	-	-	-	-	-	-	-	-	11.2	1.4	17
*elt-1(rf); daf-12(lf)*	*ceh-16*	16.5	248	-	-	-	-	-	-	-	-	17.8	2.5	19
wild-type	*dre-1*	-	-	-	-	-	-	-	-	-	-	16.0	0.0	12
*daf-12(lf)*	*dre-1*	-	-	-	-	-	-	-	-	-	-	20.9	1.5	13
*elt-1(rf)*	*dre-1*	-	-	-	-	-	-	-	-	-	-	14.3	1.6	15
*elt-1(rf); daf-12(lf)*	*dre-1*	28.6	154	-	-	-	-	-	-	-	-	24.0	4.1	20

^a^L4 Bursting Vulva, Alae Formation, and Young Adult Seam cell phenotypes were analyzed for strains of indicated genotypes and RNAi treatment. *elt-1(rf)* is the partial loss-of-function allele *ku491*. *daf-12(lf)* is the loss-of-function allele *rh61rh411*.

^b^For bursting vulva rate, the p-value for the comparison of *elt-1(ku491); daf-12(lf)* animals on empty-vector (-) RNAi vs target gene RNAi is < 0.0001 for each of *lin-28*, *hbl-1*, *lin-14*, *lin-41 and mab-10*, 0.003 for *lin-46*, 0.0002 for *lin-42*, 0.983 for *kin-20*, 0.0003 for *ceh-16*, and 0.315 for *dre-1*.

^c^For seam cell numbers, the p-value for the comparison of *elt-1(ku491);daf-12(lf)* animals on empty-vector (-) RNAi vs on target gene RNAi is <0.0001 for *lin-28*, *hbl-1*, *lin-14*, *lin-41*, *lin-46*, *lin-42*, *kin-20*, *mab-10*, *ceh-16*, and *dre-1*, and > 0.9999 for *lin-29*.

Abbreviations: e.v., empty vector; Abs, absent; Gap, gapped; Pres, present; #, seam cell number measured using the *scm*::*GFP* reporter; Std Dev, standard deviation.

Interestingly, the *daf-12(null)*-independent defect in the formation of adult alae seen in *elt-1/GATA(ku491)* single-mutant animals was not affected by knock-down of any of the genes examined. These results indicate that the defect in developmental timing in *elt-1(ku491); daf-12(rh61rh411)* double-mutant animals is likely to be within or upstream of the part of the heterochronic gene network that controls late larval stages, while the *daf-12(null)*-independent defects of *elt-1(ku491)* animals are independent of the heterochronic gene network.

### *elt-1/GATA* mutant animals have defective down-regulation of *lin-41* during the L4 stage

As shown in Figs. 1, 2, S1, and Table 1, the heterochronic phenotypes of the *elt-1/GATA(ku491)*; *daf-12(rh61rh411)* double-mutant animals are during late stages of post-embryonic development, L4 and Young Adulthood. The LIN-28 and HBL-1 proteins are known to be down-regulated at the L2 molt and during the L3 stage, respectively [8], which is mostly prior to the emergence of the heterochronic phenotypes of *elt-1(ku491); daf-12(rh61rh411)* double-mutant animals. The expression level of these genes’ mRNA at the L4 stage was found to be normally down-regulated in *elt-1/GATA(ku491)*; *daf-12(rh61rh411)* double-mutant animals (S4 Fig). Therefore, altered expression of *lin-28* and *hbl-1* is unlikely to be responsible for the phenotypes of *elt-1(ku491); daf-12(rh61rh411)* double-mutants.

**Fig 3:**
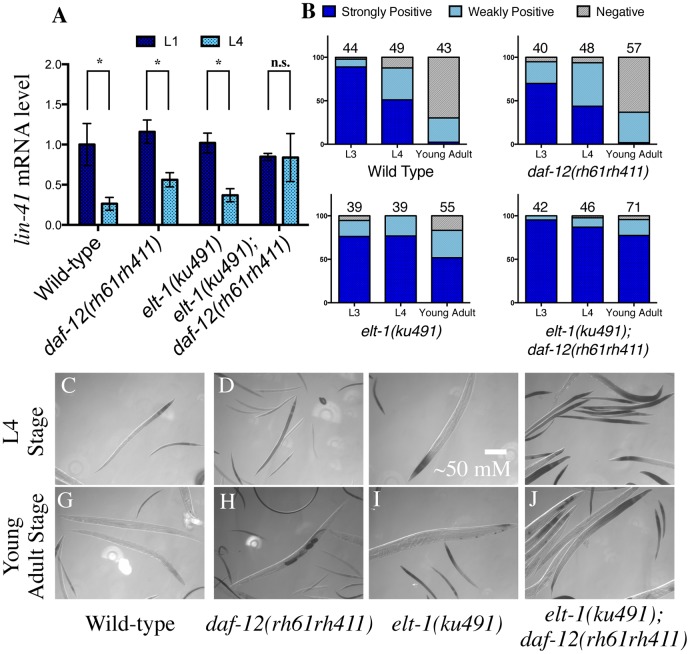
*elt-1(ku491);daf-12(rh61rh411)* double-mutant animals fail to down-regulate the heterochronic gene *lin-41* during development. A, Lin-41 mRNA is down-regulated normally during L4 in wild-type and single-mutant animals as measured with RT-qPCR, but *elt-1(ku491);daf-12(rh61rh411)* double-mutant animals continue to express it at a level indistinguishable from their L1 level. Detailed descriptive statistics and p-values in S4 Fig. B, *elt-1(ku491);daf-12(rh61rh411)* double-mutant animals fail to down-regulate a *lin-41* 3’UTR reporter. The number scored per genotype and stage are noted above each column. Representative images of X-gal stain shown at L4 (C-F) and Young Adult (G-J) stages.

The LIN-41 protein is also highly expressed during larval development, but in contrast to LIN-28 and HBL-1, it is down-regulated primarily during the L4 stage by the LET-7 miRNA [3,13]. The heterochronic phenotypes of *elt-1/GATA(ku491); daf-12(rh61rh411)* double-mutant animals are at the same developmental stage as when LIN-41 is normally down-regulated, so the dynamics of *lin-41* gene expression were examined in these mutant animals. As shown in Fig. 3A, *elt-1/GATA(ku491); daf-12(rh61rh411)* double-mutant animals fail to down-regulate the level of the *lin-41* mRNA, in contrast to animals that are wild-type or carry either mutation individually. Summary descriptive statistics and statistical analysis for all mRNA qPCR results are shown in S4 Fig.

To further examine the regulation of *lin-41* mRNA during L4 in *elt-1(ku491); daf-12(rh61rh411)* double-mutant animals, the integrated transgene *pkIs2084* was obtained and crossed into the mutant strains; *pkIs2084* contains the *beta-galactosidase* gene under the control of the pan-hypodermal *col-10* promoter and the *lin-41* 3’ untranslated region (3’UTR). This reporter has previously been shown to be down regulated in wild-type animals from the L3 stage to the L4 and young adult stages, and that this down regulation requires both LET-7 miRNA and the cofactors needed for miRNA-induced gene silencing (e.g., Argonaute) [3,44]. We found that *elt-1/GATA(ku491); daf-12(rh61rh411)* double-mutant animals fail to down-regulate the reporter correctly (Fig. 3B-J), indicating that those animals are defective in the negative regulation of the *lin-41* 3’UTR that normally occurs during the L4 stage. These results suggest that *elt-1* normally contributes to developmental timing, at least in part, by promoting the down-regulation of *lin-41* expression during L4, and that it may do so by promoting the expression of LET*-7*.

### *elt-1/GATA* normally promotes the expression of the developmental timing miRNAs LET-7, miR-48, miR-84, and miR-241

**Fig 4:**
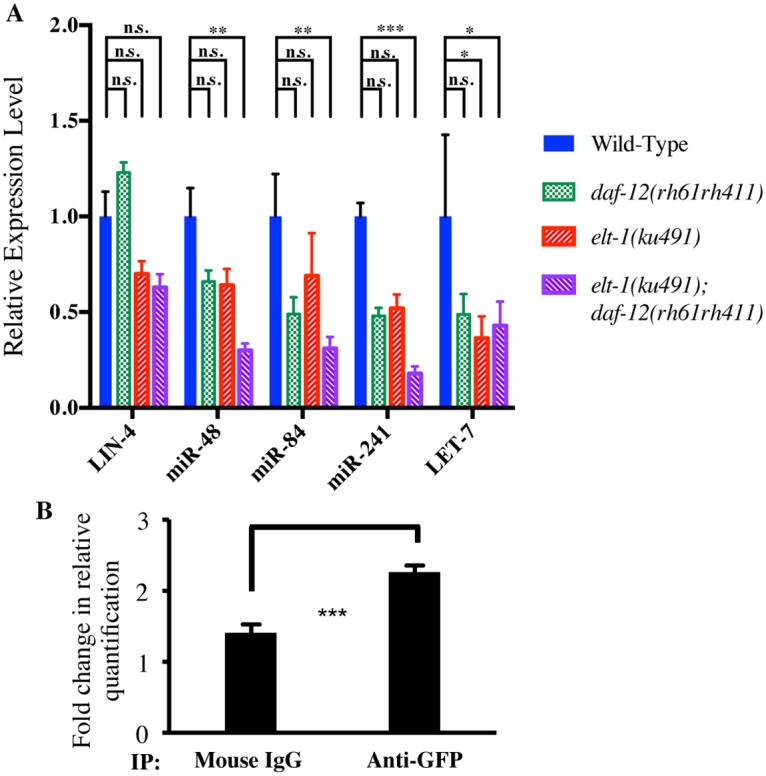
LET-7 family miRNAs are decreased in *elt-1(ku491);daf-12(rh61rh411)* double-mutant animals and ELT-1 binds to the *let-7* promoter *in vivo*. A. ELT-1 and DAF-12 redundantly regulate LET-7, miR-48, miR-84, and miR-241. The expression level of target miRNAs was determined by RT-qPCR using synchronous L4 animals. Graphs show mean ± standard error. Detailed descriptive statistics and p-values are listed in S2 Table. B, ChIP-qPCR data showing enrichment of ELT-1 binding to a region in the *let-7* promoter. Samples from a strain expressing an ELT-1::GFP fusion transgene were subject to immunoprecipitation using either control IgG or anti-GFP antibody. The primers were specific to a region 1.7kb upstream of the *let-7* transcription start site (Ch. X, 14747074 to 14747179). Graphs show mean ± standard error of triplicate experiments. ELT-1 also binds to promoters of other *let-7* family genes (See Table 3). p-values: *, < 0.05; **, < 0.01; ***, < 0.001; ****, < 0.0001.

The LET-7 family of miRNAs (miR-48, miR-84, and miR-241) have previously been shown to be expressed during or near the L2 molt to promote developmental progression [27,28], while LET-7 is expressed primarily during L4 and required for the L4-to-Adult transition, largely by down-regulating *lin-41* mRNA [3,13]. The expression of the LET-7, miR-48, miR-84, and miR-241 miRNAs was therefore examined during the L4 stage in *elt-1(ku491); daf-12(rh61rh411)* double-mutant animals using RT-qPCR. As shown in Fig. 4A, *elt-1(ku491); daf-12(rh61rh411)* double-mutant animals have deficient expression of LET-7 and each of the LET-7 family of miRNAs (miR-48, miR-84, and Mir-241). The heterochronic miRNA LIN-4 was also analyzed, although its expression is initiated at early larval stages to drive the L1-to-L2 transition, and was therefore expected to be normal in these mutants. Summary descriptive statistics and statistical analysis results are in S2 Table.

**Table 3:** Analysis of modENCODE ChIP-Sequencing with ELT-1::GFP for selected genes.

Putative Target Gene					ChIP: ELT-1, L2 Stage					ChIP: ELT-1, L3 Stage				
Name	Position				TF Binding Site^a^			Rank^b^	IDR Value	TF Binding Site^a^			Rank^c^	IDR Value
	Ch.	Start	Stop	Strand	Ch.	Start	Stop			Ch.	Start	Stop		
*lin-28*	I	8,407,802	8,410,804	Anti.	-	-	-	-	-	I	8,409,817	8,410,200	1991	3.719248398
										I	8,411,201	8,411,584	1059	3.719248398
*lin-41*	I	9,334,850	9,342,496	Anti.	I	9,332,441	9,332,921	175	2.830588669	I	9,332,518	9,332,792	61	3.719248398
					I	9,343,966	9,344,446	302	2.830588669	I	9,334,389	9,334,773	252	3.719248398
										I	9,343,706	9,344,090	1309	3.719248398
*lin-4*	II	5,902,254	5,902,347	Sense	-	-	-	-	-		-	-	-	-
*lin-29*	II	11,917,642	11,935,343	Anti.	-	-	-	-	-		-	-	-	-
*elt-1*	IV	9,615,156	9,620,141	Sense	IV	9,609,064	9,609,544	132	2.830588669	IV	9,607,039	9,607,423	892	3.719248398
					IV	9,609,787	9,610,267	182	2.830588669	IV	9,607,979	9,608,239	30	3.719248398
					IV	9,613,054	9,613,534	116	2.830588669	IV	9,609,179	9,609,410	95	3.719248398
					IV	9,614,144	9,614,377	36	2.830588669	IV	9,610,027	9,610,411	2272	3.719248398
					IV	9,615,365	9,615,978	1	2.830588669	IV	9,611,032	9,611,250	183	3.719248398
					IV	9,617,254	9,617,734	148	2.830588669	IV	9,611,608	9,611,992	317	3.719248398
					IV	9,618,016	9,618,496	88	2.830588669	IV	9,613,176	9,613,307	116	3.719248398
					IV	9,618,941	9,619,094	42	2.830588669	IV	9,614,092	9,614,503	3	3.719248398
										IV	9,615,323	9,615,967	1	3.719248398
										IV	9,617,377	9,617,686	69	3.719248398
										IV	9,618,060	9,618,399	13	3.719248398
										IV	9,618,953	9,619,135	152	3.719248398
*mir-48*	V	14,364,412	14,364,509	Anti.	-	-	-	-	-	V	14,366,523	14,366,907	1247	3.719248398
*mir-241*	V	14,366,188	14,366,283	Anti.	-	-	-	-	-	V	14,368,343	14,368,727	2458	3.719248398
*hbl-1*	X	5,822,277	5,827,691	Sense	-	-	-	-	-	X	5,820,110	5,820,494	2507	3.719248398
										X	5,828,024	5,828,408	1205	3.719248398
*daf-12*	X	10,644,407	10,666,869	Sense	-	-	-	-	-	-	-	-	-	-
*let-7*	X	14,743,590	14,745,321	Anti.	-	-	-	-	-	X	14,746,915	14,747,299	1366	3.719248398
										X	14,753,494	14,753,878	1121	3.719248398
*mir-84*	X	16,022,404	16,022,478	Anti.	-	-	-	-	-	X	16,024,462	16,024,846	1218	3.719248398

^a^Transcription factor binding sites within approximately 5kb of target genes are listed. “-”is used to denote genes without putative transcription factor binding sites nearby.

^b^Out of 327 identified TF binding sites,

^c^Out of 2546 identified TF binding sites. Abbreviations: TF Binding Site, Transcription Factor Binding Site; Ch., Chromosome; IDR, Irreproducible Discovery Rate; Anti., antisense.

### The ELT-1 protein binds near the sequences encoding the *let-7* miRNA family *in vivo*

A recent study examined the binding sites of a wide range of *C*. *elegans* transcription factors, including of ELT-1, using Chromatin Immunoprecipitation followed by high-throughput sequencing [45]. Examining their peak-calls near selected heterochronic genes (summarized in Table 3), we identified that ELT-1 binds to sites in the likely promoter regions for the DNA sequences encoding the *let-7* family miRNAs during the L3 stage, while binding was not detected during the L2 stage. L4 stage-specific ChIP of ELT-1 was not included in this report [44]. To partially replicate their genome-wide ChIP-seq data for ELT-1, we performed qPCR following ChIP using L3-L4 stage-enriched worms. We designed qPCR primers based on 9 putative GATA transcription-factor binding sites within three kilobases 5’ of the transcription start site of *let-7* gene. Among them, one primer set showed statistically-significant enrichment of ELT-1 binding (Fig. 4B). The genomic region corresponding to this primer set (Ch. X: 14,747,074 to 14,747,179; ~1.7 kb upstream of the transcription start site) overlapped with a binding site found in the modENCODE ChIP-seq data (Ch. X: 14,746,915 to 14,747,299; Table 3), supporting their finding that ELT-1 directly regulates the transcription of the *let-7* gene.

## Discussion

The study of postembryonic developmental timing in *C*. *elegans* has made important contributions to our understanding of the mechanisms of temporal developmental control in multicellular animals, including the initial discovery of miRNAs and of their role in the temporal regulation of key heterochronic genes’ expression [1–3,5]. Given that stage-specific expressions of these miRNAs controls the dynamic state of the heterochronic gene network, understanding the regulation of the expression of these miRNAs is an important problem with significant gaps in our current understanding [7]. In this study, we used a genetic enhancer screen to identify the GATA transcription factor ELT-1 as a new heterochronic gene and have shown that it contributes to developmental timing by providing positive regulation of the expression of the developmental timing miRNAs LET-7, miR-48, miR-84, and miR-241.

In *C*. *elegans*, the GATA transcription factor *elt-1* has previously been shown to be required for formation of the hypodermis during embryonic development [46] and for the maintenance of cell fate in the seam-cell lineage and adult alae formation during post-embryonic development [35–37]. In this paper, analysis of a non-*null* allele of *elt-1* identified from a random mutagenesis screen demonstrates that *elt-1* is heterochronic gene that acts in parallel to the nuclear-hormone receptor *daf-12* to provide essential regulation of late-larval stage-specific cell fates. Therefore, a genetic screen in a sensitized background with isolation of a partial loss-of-function allele allowed us to genetically separate the post-embryonic roles for *elt-1* from its role in embryonic development; the role of *elt-1/GATA* in developmental timing was previously masked due to both pleiotropism and genetic redundancy.

The phenotype of *elt-1(ku491); daf-12(rh61rh411)* double-mutant animals is during late developmental stages, with seam cell proliferation during the L4 and Young Adult stages and an L4 bursting vulva phenotype (Figs. 1–2, Table 1). Epistasis analysis (Table 2) shows that the heterochronic phenotypes of *elt-1(ku491); daf-12(rh61rh411)* mutants require the function of the heterochronic gene network. In addition, the seam-cell proliferation defects, but not bursting-vulva phenotype, can be suppressed by knock-down of genes previously shown to regulate seam-cell maintenance or fate downstream of the heterochronic gene network [40–42]. For genes with partial suppression, such as *mab-10* and *kin-*20, this would seem most likely due to premature adoption of later cell fates (suppressing the seam-cell phenotype) but without precocious expression of LET-7 family miRNAs (to suppress the bursting-vulva phenotype). These data suggest that the molecular defect in *elt-1(ku491); daf-12(rh61rh411)* double-mutant animals may be in the expression of an L4-specific regulatory factor. During the L4 stage, the major target of heterochronic miRs is the *lin-41* mRNA [3,8], and the down regulation of *lin-41* mRNA that occurs during that stage has previously been shown to require LET-7 [13]. Indeed, the RT-qPCR analysis of *lin-41* mRNA levels during L4 presented here (Fig. 3A) is consistent with a defect in *elt-1(ku491); daf-12(rh61rh411)* double-mutant animals at the level of an L4-stage-specific regulatory factor that negatively regulates the *lin-41* mRNA.

This L4-stage-specific regulatory factor may, in fact, be the LET-7 miRNA, as *elt-1(ku491); daf-12(rh61rh411)* double-mutant animals fail to down-regulate the *lacZ*::*lin-41* 3’UTR reporter during L4 (Fig. 3B-J) and have decreased expression of the LET-7 miR as measured by RT-qPCR (Fig. 4A). The regulation of *lin-41* mRNA by LET-7 has previously been shown to be essential for L4-stage-specific developmental progression [3] and the phenotype of *elt-1(ku491); daf-12(rh61rh411)* double-mutant animals is consistent with that expected from reduced expression of LET-7. These data indicate that *elt-1* promotes LET-7 expression during the L4 stage, a novel and unexpected finding. While the LET-7 miR qPCR data (Fig. 4A) are limited by noise likely intrinsic to the time of the measurement, all of the data, including the phenotypes, mRNA qPCR and lacZ staining, are consistent with the interpretation that *elt-1* and *daf-12* each provide redundant regulation of LET-7 that is required for its L4-stage-specific expression.

**Fig 5:**
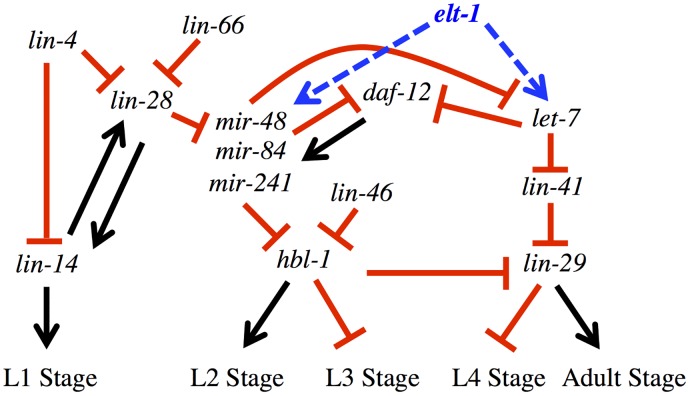
*elt-1/GATA* promotes the expression of multiple miRNAs that have key roles in the developmental timing regulatory pathway. Proposed role for *elt-1/GATA* in the heterochronic gene regulatory network. Arrows and T-bars indicated positive and negative regulatory relationships, respectively. Model adapted from Resnick, McCulloch, and Rougvie (2010) [7].

However, the decreased expression of LET-7 alone is unlikely to be the sole cause of the developmental timing phenotypes (Figs. 1, 2A, S1, and Table 1) or defective L4-stage down-regulation of *lin-41* mRNA seen in the *elt-1(ku491); daf-12(rh61rh411)* double-mutant animals (Fig. 3A-J), as LET-7 is expressed at a similarly-decreased level in the *elt-1(ku491); daf-12(rh61rh411)* double mutants as in each of the single-mutant strains (Fig. 4A), which lack strong developmental timing phenotypes and correctly down-regulate *lin-41* mRNA (Fig. 3A-B). However, the three LET-7 family miRNAs (miR-48, miR-84, and miR-241) all have a statistically-significant decrease in their expression during the L4 stage in the *elt-1(ku491); daf-12(rh61rh411)* double-mutant animals but not in either single-mutant, which likely accounts for the differences in the phenotypes and data. This suggests that both *daf-12* and *elt-1* promote the expression of miR-48, miR-84, and miR-241, but that this regulation is highly redundant, so that either transcription factor alone is sufficient to promote sufficient expression of the miRs to prevent a gross phenotype in the single-mutant strains, despite defective expression of LET-7 in those single-mutant animals. Fig. 5 is a proposed model for the role of *elt-1* in the regulation miRNA expression in the heterochronic gene network. These data are also consistent with previous studies showing that *daf-12* regulates developmental progression [25,26] and the expression of miR-48, miR-84, miR-241 and LET-7 at the L2 molt [28] and L3 stages [27].

A recent study of transcription factor binding sites in *C*. *elegans* included ELT-1 [45], and analysis of their data (Table 3) shows that the ELT-1 protein binds the likely promoter region of the DNA sequences encoding all members of the LET-7 miRNA family (*mir-48*, *mir-84*, *mir-241*, *let-7)*, supporting the idea that ELT-1 directly regulates the transcription of the *let-7* family miRNAs during late larval development. Our independent analysis confirmed ELT-1 binding in the *let-7* promoter region (Fig. 4B). This binding site is 1.6kb upstream of the previously known temporal regulatory element (TRE) [32], so it would be of interest to determine the relationship between the ELT-1 binding site and the TRE in the regulation of *let-7* expression.

ELT-1 also has binding sites near the *lin-41*, *lin-28*, and *hbl-1* genes during the L3 stage, but it is unclear whether these sites are functionally significant, as *lin-28* and *hbl-1* appear to be normally expressed in the *elt-1(ku491)* mutants, and the abnormal expression of *lin-41* in *elt-1* mutants appears to be due to defective down-regulation of its 3’UTR. Intriguingly, ELT-1 protein is expressed in the hypodermis during embryonic development [46] and seam cells during post-embryonic development [36,37], but it remains unclear why it only promotes the expression of LET-7 family miRNAs during late larval stages, rather than throughout development. Perhaps it interacts with other stage-specific transcription factors, undergoes stage-specific post-translational modifications, or its binding sites near those genes are masked or unmasked in a stage-specific manner.

In summary, the *elt-1/GATA(ku491)* allele described in this study has uncovered a function for *elt-1* in regulating miRNA expression and developmental timing that was previously masked by pleiotropism and genetic redundancy. This forcefully supports the idea that the robust expression of key developmental timing genes comes from regulation by parallel and redundant regulatory mechanisms. Similar mechanisms of robustness may also be important in regulating miRNA expression in other organisms during critical developmental transitions, such as in the differentiation of stem cells and in the maintenance of differentiated cell states.

## Materials and Methods

### Nematode methods

Worms were maintained at 20°C and handled as previously described [47]. Additional information can be found in the S1 Text.

### Phenotype assessment

Phenotypes were scored, and feeding RNAi was performed starting at the L1 stage, as previously described [1,2,33,48]. Statistical comparisons of seam cell phenotypes were performed with Prism 6 using one-way ANOVAs with p-values calculated with Bonferroni’s multiple comparisons method. For L4 bursting vulva rate, data was analyzed with Prism 6 and p-values were calculated by 2-tailed binomial t-test. Additional information can be found in the S1 Text.

### Quantitative measurement of mRNA and microRNA expression levels

Stage-specific samples were prepared by picking individual worms from mixed-stage plates based on gross appearance and vulval morphology; 50–100 animals were collected per sample. RT-qPCR was performed as previously described [33,49,50] with normalization to *eef-2*. Expression of miRNAs was measured from the same RNA samples using TaqMan miRNA assay kits (Invitrogen Corp.) with normalization to the snoRNA U18, as recommended by the manufacturer. Statistical analysis was performed with Prism 6 (GraphPad) using 2-way ANOVA with p-values calculated using Sidak’s multiple comparisons test. Additional information can be found in the S1 Text.

### Expression of an ***in vivo*** reporter of regulation on the ***lin-41*** 3’ untranslated region

The *pkIs2084* integrated reporter [3,44] was obtained and crossed into the indicated strains. Staining for lacZ activity was performed as described [51]; saturated staining at any point in the animal was scored as strong positive, visible but unsaturated staining as weak positive, and undetectable staining as negative.

### Analysis of modENCODE ELT-1::GFP ChIP-sequencing data

modENCODE data sets with stage-specific ChIP-sequencing of an ELT-1::GFP array in strain OP354 at the L2 and L3 stage (modENCODE data coordinating center identifying numbers 4632 and 3843, respectively) were obtained from the website (listed below) and examined for transcription factor sites near selected genes [45,52,53]. The Blacklist Filtered Peak Calls file was used for analysis and is available online at https://www.encodeproject.org/comparative/regulation/#Wormset7.

### Chromatin Immunoprecipitation (ChIP) analysis

OP354 strain (*unc-119(tm4063*); wgIs354 [elt-1::TY1::EGFP::3xFLAG + *unc-119(+)*]) was synchronized by bleaching and collected at the L3-L4 stages. The ChIP experiment was performed as described previously [54] with minor modifications. Briefly, paraformaldehyde-fixed chromatin was immunoprecipitated with either mouse IgG (Jackson immunoresearch) complexed with Protein G beads (GE healthcare) or TrapA GFP beads (Chromotek). Following extraction of the immunoprecipitated DNA, qPCR was performed according to the manufacturer’s instruction (Bioneer). Primers specific to *let-7* promoter region are as follows (5’-3’): forward primer, TCTCACTGTGTGTCAGCCG, and reverse primer, TGCTGACGTACTACCGGTGC5. The result was normalized to the level of 3’Untranlsated Region of *let-7* gene completed from the same immune complexes using the following primers (5’-TCGATCTCTGTCCGCTTTGAAAC-3’, 5’-CAGGAGGTGAAGAACGAGCA-3’).

## Supporting Information

S1 Fig L1-L2 seam cells phenotypes and summary statistics. A, Seam cells numbers in wild-type, *daf-12(rh61rh411)*, *elt-1(ku491)*, *and elt-1(ku491); daf-12(rh61rh411)* strains at the L1 and L2 stages. B, Summary statistics for seam cell numbers at all stages. Results for L4 and Young Adult stages are presented in Fig. 1 and main text. At the L1, L2, and L3 stages, the only statically-significant difference in the number of seam cells between the strains examined is at L3 between wild-type and *elt-1(ku491); daf-12(rh61rh411)* double-mutants (p-value, 0.0265). At L4, the p-value for each single mutant compared to wild-type is not significant, the comparison of *elt-1(ku491)* to *daf-12(rh61rh411)* has a p-value of 0.0386, and the comparison of wild-type or single mutants with the *elt-1(ku491); daf-12(rh61rh411)* double-mutants is less than 0.0001. At the young-adult stage, the p-value for all comparisons with the *elt-1(ku491); daf-12(rh61rh411)* double-mutants is less than 0.0001, and all other comparisons are not statistically significant (*elt-1(ku491)* vs *daf-12(rh61rh411)* has a p-value of 0.0656).
(TIFF)

S2 Fig Sequence alignment of ELT-1 with related proteins. Alignment of *C. elegans* ELT-1 with related proteins showing the conserved Zn-finger DNA binding domains. ELT-1 proline^298^ is at alignment position number 485.
(TIFF)

S3 Fig Structure of *M*. *musculus* GATA1 Zn-finger domains bound to palindromic DNA highlighting residue conserved with *C*. *elegans* ELT-1 proline^298^. A-C, PDB structure 3VD6 with yellow arrow highlights amino acid residue corresponding to *C. elegans* ELT-1 proline^298^.
(TIFF)

S4 Fig Analysis of key heterochronic genes’ mRNA expression during development. A, RT-qPCR of *lin-28* and *hbl-1* mRNA shows normal down-regulation in *elt-1(ku491);daf-12(rh61rh411)* double-mutants. B, Descriptive statistics of RT-qPCR performed for mRNA of indicated genes. C, P-values for L1 vs L4 expression levels of the indicated genes for each strain.
(TIFF)

S1 Table Phenotypes of elt-1 alleles from million-mutation project. ^a^p-values are for comparison to the *daf-12*(rh61rh411) strain.
(DOCX)

S2 Table Analysis of microRNA qPCR data. A, Summary statistics for qPCR data. B, Statistical comparisons of qPCR data.
(DOCX)

S1 Text Supplemental materials and methods, supplemental references. (DOCX)
