# Correction: The GATA Factor *elt-1* Regulates *C*. *elegans* Developmental Timing by Promoting Expression of the *let-7* Family MicroRNAs

**DOI:** 10.1371/journal.pgen.1005257

**Published:** 2015-05-13

**Authors:** The PLOS Genetics Staff

There are errors in Table 1. The trans-heterozygous genotypes under ‘***etl-1* genotype**’ are not displayed in the standard way; *ku491*+ should be *ku491*/+, *ok1002*+ should be *ok1002*/+ and *ku491* *ok1002* should be *ku491*/*ok1002*. The correct table is displayed underneath.

**Table 1:** *elt-1* mutants have an L4-stage bursting vulva and defective alae formation and *elt-1(ku491)* is a partial loss-of- function allele.

Strain	RNAi	L4 Bursting Vulva		L4 Molt Alae				Young Adult Seam Cells		
		%	n	Absent (%)	Gapped (%)	Present (%)	n	SCM	Std Dev	n
wild-type	Empty vector^a^	0.0	245	0.0	0.0	100.0	27	16.0	0.4	27
*daf-12(rh61rh411)*	Empty vector^a^	0.0	183	0.0	0.0	100.0	24	18.1	1.6	24
*elt-1(ku491)*	Empty vector^a^	3.6	169	88.4	3.8	7.6	26	12.9	2.2	15
*elt-1(ku491);daf-12(rh61rh411)*	Empty vector^a^	55.1	198	88.8	0.0	11.1	18	39.7	9.8	29
*wild-type*	*elt-1*	48.7	117	100.0	0.0	0.0	21	0.5	0.8	21
*daf-12(rh61rh411)*	*elt-1*	56.7	90	90.0	5.0	5.0	20	0.2	0.4	20
*elt-1(ku491)*	*elt-1*	76.3	114	100.0	0.0	0.0	27	1.3	1.2	27
*elt-1(ku491);daf-12(rh61rh411)*	*elt-1*	94.8	231	96.4	3.6	0.0	28	2.0	2.2	28
***elt-1* genotype** ^b^	***daf-12* genotype**									
*ku491/+*	*Wild-type*	0.0	60	0.0	0.0	100.0	19	15.9	0.3	18
*ku491/+*	*rh61rh411*	0.0	57	0.0	0.0	100.0	18	18.3	1.8	18
*ok1002/+*	*Wild-type*	0.0	92	0.0	0.0	100.0	18	16.0	0.3	18
*ok1002/+*	*rh61rh411*	0.0	78	0.0	0.0	100.0	17	18.2	1.9	17
*ku491/ok1002*	*Wild-type*	1.9	52	84.0	16.0	0.0	25	9.4^c^	2.0	25
*ku491/ok1002*	*rh61rh411*	65.9	41	100.0	0.0	0.0	20	18.2^d^	6.6	20

^a^Phenotypes on empty-vector control RNAi were similar to the standard *E*. *coli* strain OP50.

^b^An allele of *elt-1(ku491)* linked to mutations in *unc-24* and *dpy-20* was used for these strains.

^c^For *elt-1(ku491)* animals vs *elt-1(ku491)-over-elt-1(null)* animals at the young adult stage, the p-value for the comparison of seam-cell numbers is 0.0340

^d^For *elt-1(ku491);daf-12(rh61rh411)* animals vs *elt-1(ku491)-over-elt-1(null);daf-12(rh61rh411)* animals at the young adult stage, the p-value for the comparison of seam-cell numbers is < 0.0001.
