# Supplementary figures and images for: The GATA Factor elt-1 Regulates C. elegans Developmental Timing by Promoting Expression of the let-7 Family MicroRNAs
Source: PLoS Genet. 2015 Mar 27;11(3):e1005099. doi: 10.1371/journal.pgen.1005099 (PMC4376641; doi:10.1371/journal.pgen.1005099)

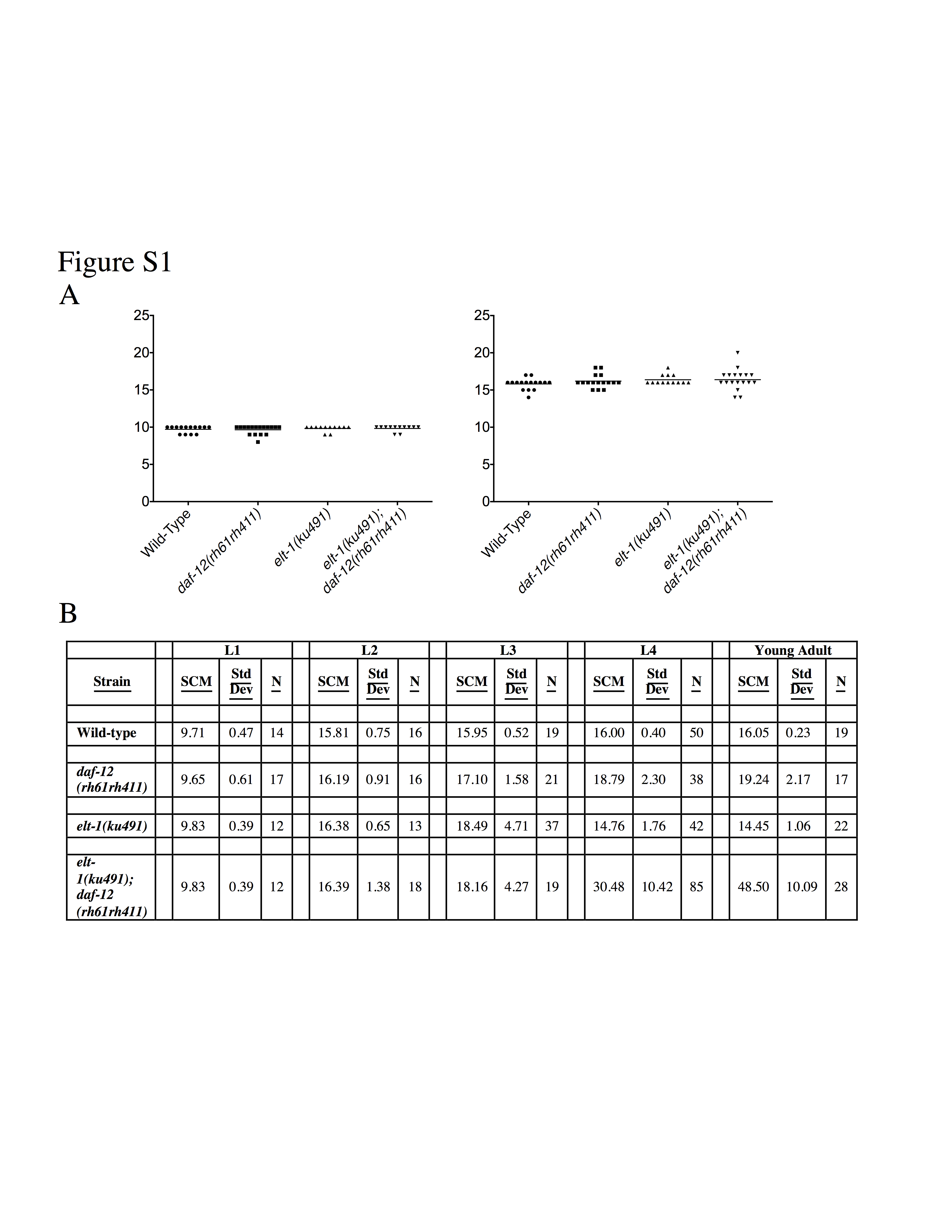

Supplement: S1 Fig — A, Seam cells numbers in wild-type, daf-12(rh61rh411), elt-1(ku491), and elt-1(ku491); daf-12(rh61rh411) strains at the L1 and L2 stages. B, Summary statistics for seam cell numbers at all stages. Results for L4 and Young Adult stages are presented in Fig. 1 and main text. At the L1, L2, and L3 stages, the only statically-significant difference in the number of seam cells between the strains examined is at L3 between wild-type and elt-1(ku491); daf-12(rh61rh411) double-mutants (p-value, 0.0265). At L4, the p-value for each single mutant compared to wild-type is not significant, the comparison of elt-1(ku491) to daf-12(rh61rh411) has a p-value of 0.0386, and the comparison of wild-type or single mutants with the elt-1(ku491); daf-12(rh61rh411) double-mutants is less than 0.0001. At the young-adult stage, the p-value for all comparisons with the elt-1(ku491); daf-12(rh61rh411) double-mutants is less than 0.0001, and all other comparisons are not statistically significant (elt-1(ku491) vs daf-12(rh61rh411) has a p-value of 0.0656). (TIFF) [file pgen.1005099.s001.tiff]

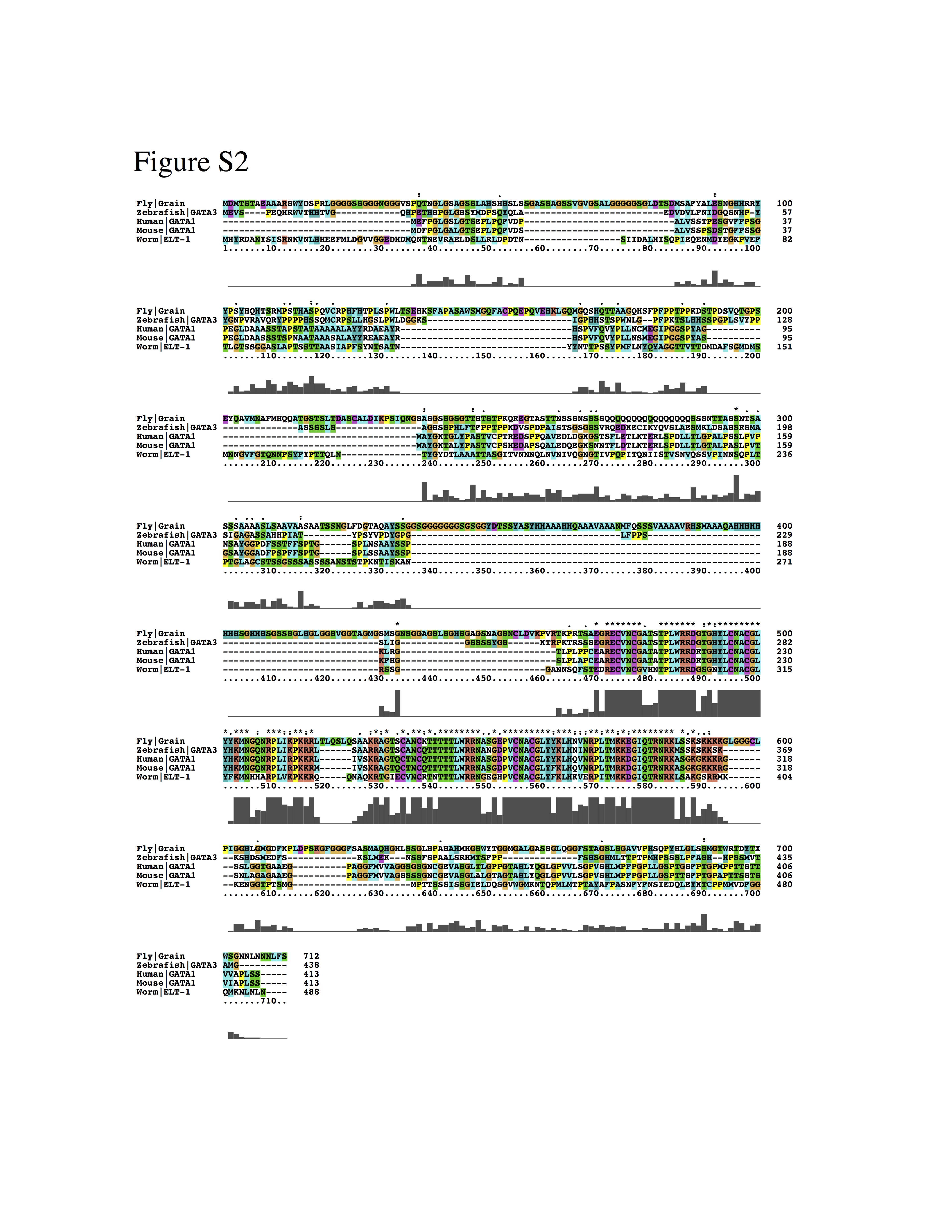

Supplement: S2 Fig — Alignment of C. elegans ELT-1 with related proteins showing the conserved Zn-finger DNA binding domains. ELT-1 proline298 is at alignment position number 485. (TIFF) [file pgen.1005099.s002.tiff]

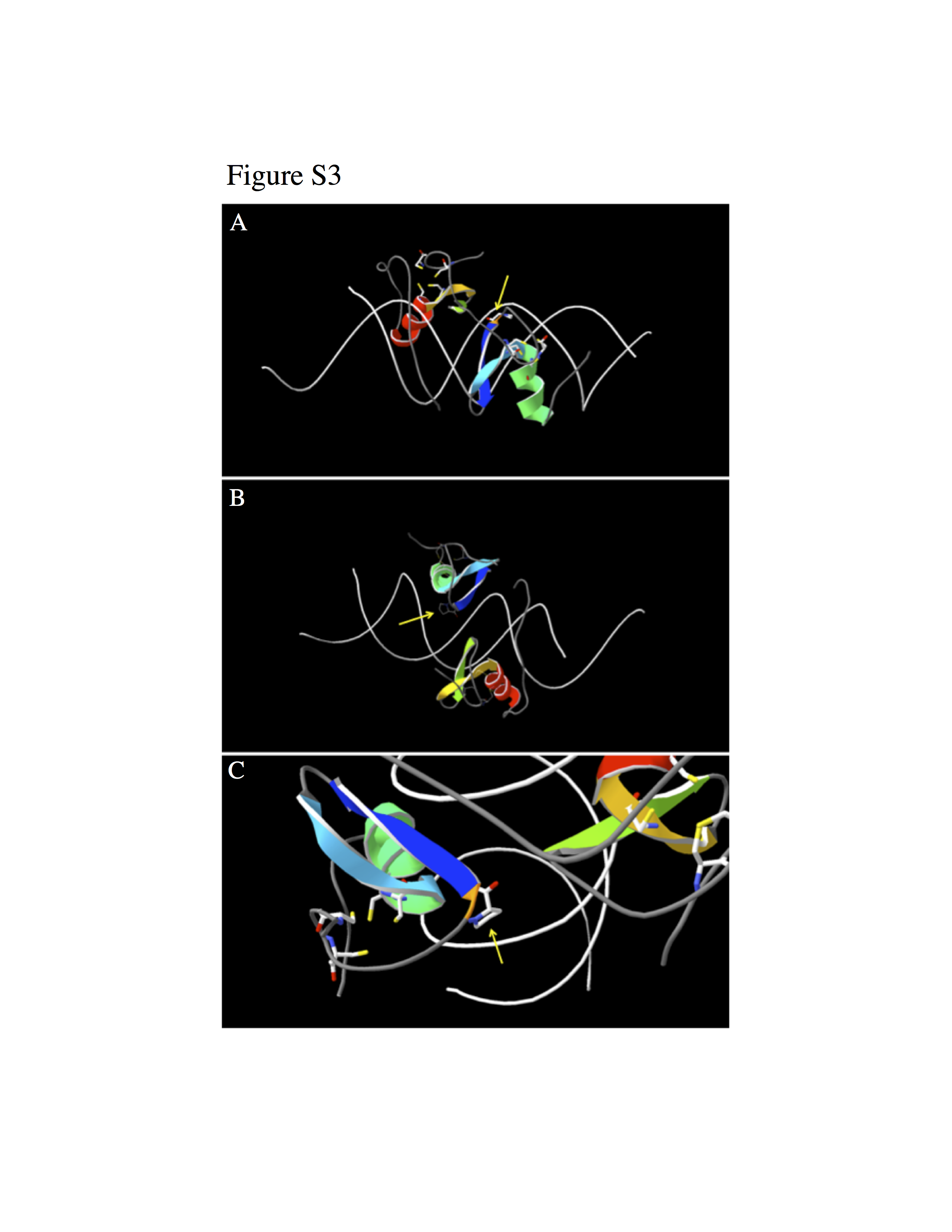

Supplement: S3 Fig — A-C, PDB structure 3VD6 with yellow arrow highlights amino acid residue corresponding to C. elegans ELT-1 proline298. (TIFF) [file pgen.1005099.s003.tiff]

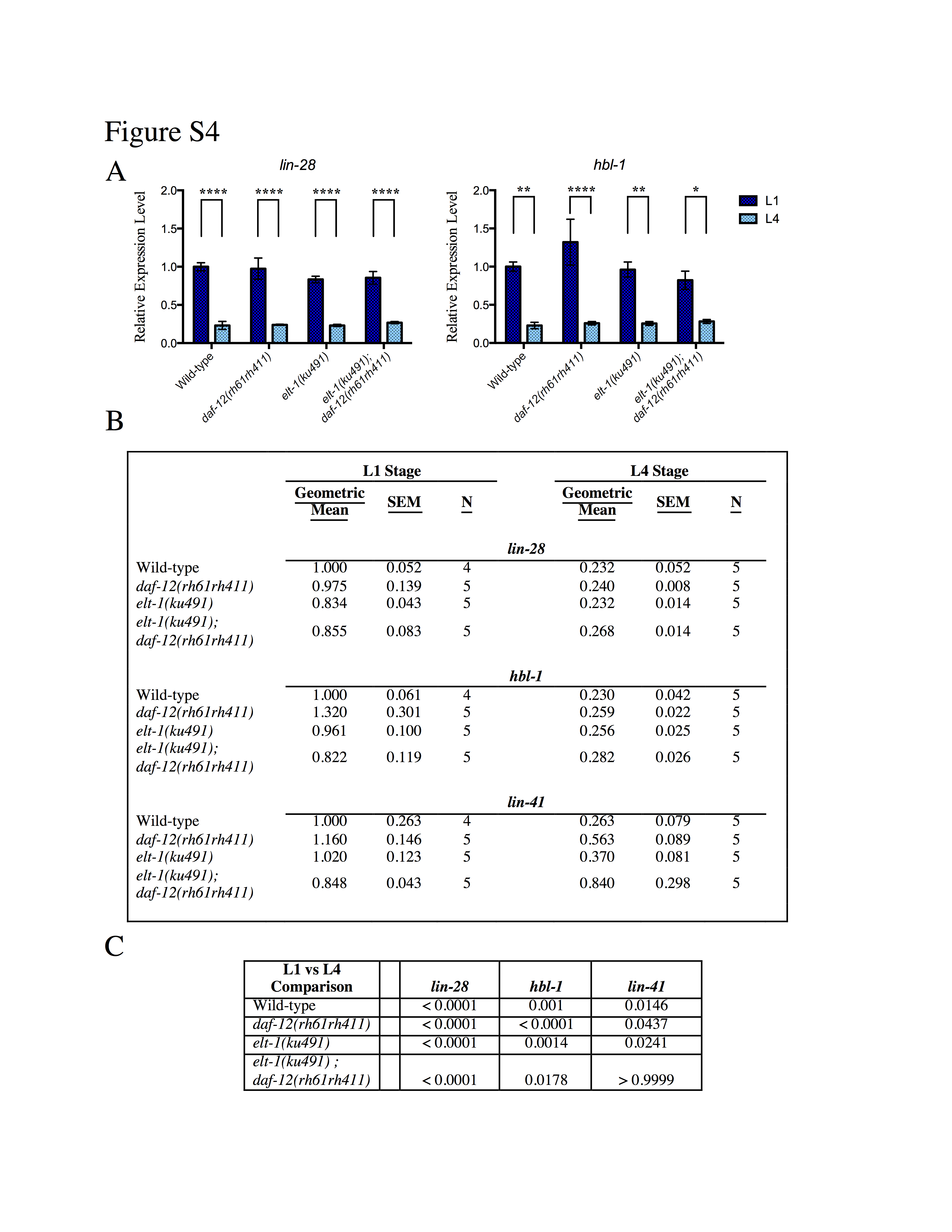

Supplement: S4 Fig — A, RT-qPCR of lin-28 and hbl-1 mRNA shows normal down-regulation in elt-1(ku491);daf-12(rh61rh411) double-mutants. B, Descriptive statistics of RT-qPCR performed for mRNA of indicated genes. C, P-values for L1 vs L4 expression levels of the indicated genes for each strain. (TIFF) [file pgen.1005099.s004.tiff]
