# Supplementary material for: The GATA Factor elt-1 Regulates C. elegans Developmental Timing by Promoting Expression of the let-7 Family MicroRNAs
Source: PLoS Genet. 2015 Mar 27;11(3):e1005099. doi: 10.1371/journal.pgen.1005099 (PMC4376641; doi:10.1371/journal.pgen.1005099)
Supplement: S1 Table — ap-values are for comparison to the daf-12(rh61rh411) strain. (DOCX) [file pgen.1005099.s005.docx]

**Table S1**

| ELT-1 Protein |  | *elt-1* genotype | *daf-12* genotype |  | L4 Bursting Vulva | |  | L4 Molt Alae | | | |  | Young Adult Seam Cells | | | |
| --- | --- | --- | --- | --- | --- | --- | --- | --- | --- | --- | --- | --- | --- | --- | --- | --- |
|  |  |  |  |  | % | n |  | Absent (%) | Gapped (%) | Present (%) | n |  | SCM | Std Dev | N | p-value^a^ |
|  |  |  |  |  |  |  |  |  |  |  |  |  |  |  |  |  |
| wild-type |  | wild-type | *rh61rh411* |  | < 1% | many |  | 0 | 0 | 100 | 24 |  | 19.2 | 2.2 | 17 | - |
| G387E |  | *gk636276* | *rh61rh411* |  | < 1% | many |  | 0 | 0.0 | 100 | 13 |  | 18.5 | 1.6 | 13 | 0.3147 |
| T410I |  | *gk540167* | *rh61rh411* |  | < 1% | many |  | 0 | 0.0 | 100 | 13 |  | 23.7 | 2.3 | 13 | < 0.0001 |
| G341E |  | *gk805317* | *rh61rh411* |  | < 1% | many |  | 0 | 6.7 | 93.3 | 15 |  | 23.1 | 2.1 | 15 | < 0.0001 |
| G414R |  | *gk678013* | *rh61rh411* |  | < 1% | many |  | 0 | 0.0 | 100 | 13 |  | 19.5 | 1.8 | 13 | 0.6758 |
| S275L |  | *gk914718* | *rh61rh411* |  | < 1% | many |  | 0 | 0.0 | 100 | 12 |  | 17.6 | 1.8 | 12 | 0.0356 |
