# Supplementary material for: The GATA Factor elt-1 Regulates C. elegans Developmental Timing by Promoting Expression of the let-7 Family MicroRNAs
Source: PLoS Genet. 2015 Mar 27;11(3):e1005099. doi: 10.1371/journal.pgen.1005099 (PMC4376641; doi:10.1371/journal.pgen.1005099)
Supplement: S2 Table — A, Summary statistics for qPCR data. B, Statistical comparisons of qPCR data. (DOCX) [file pgen.1005099.s006.docx]

**Table S2**

A

|  | **Geometric Mean** | **SEM** | **N** |
| --- | --- | --- | --- |
|  |  |  |  |
|  | ***lin-4*** | | |
| Wild-type | 1.000 | 0.130 | 5 |
| *daf-12(rh61rh411)* | 1.235 | 0.053 | 5 |
| *elt-1(ku491)* | 0.702 | 0.067 | 5 |
| *elt-1(ku491);*  *daf-12(rh61rh411)* | 0.625 | 0.068 | 5 |
|  |  |  |  |
|  | ***mir-48*** | | |
| Wild-type | 1.000 | 0.149 | 5 |
| *daf-12(rh61rh411)* | 0.662 | 0.058 | 5 |
| *elt-1(ku491)* | 0.642 | 0.086 | 5 |
| *elt-1(ku491);*  *daf-12(rh61rh411)* | 0.305 | 0.036 | 5 |
|  |  |  |  |
|  | ***mir-84*** | | |
| Wild-type | 1.000 | 0.222 | 5 |
| *daf-12(rh61rh411)* | 0.492 | 0.088 | 5 |
| *elt-1(ku491)* | 0.688 | 0.223 | 5 |
| *elt-1(ku491);*  *daf-12(rh61rh411)* | 0.305 | 0.061 | 5 |
|  |  |  |  |
|  | ***mir-241*** | | |
| Wild-type | 1.000 | 0.071 | 5 |
| *daf-12(rh61rh411)* | 0.476 | 0.042 | 5 |
| *elt-1(ku491)* | 0.521 | 0.073 | 5 |
| *elt-1(ku491);*  *daf-12(rh61rh411)* | 0.184 | 0.036 | 5 |
|  |  |  |  |
|  | ***let-7*** | | |
| Wild-type | 1.000 | 0.427 | 5 |
| *daf-12(rh61rh411)* | 0.489 | 0.107 | 5 |
| *elt-1(ku491)* | 0.364 | 0.113 | 5 |
| *elt-1(ku491);*  *daf-12(rh61rh411)* | 0.430 | 0.125 | 5 |

B

| **Comparison** | | ***lin-4*** | ***mir-48*** | ***mir-84*** | ***mir-241*** | ***let-7*** |
| --- | --- | --- | --- | --- | --- | --- |
| Wild-Type | *daf-12(rh61rh411)* | 0.8348 | 0.4575 | 0.0797 | 0.0702 | 0.0784 |
| Wild-Type | *elt-1(ku491)* | 0.6017 | 0.3901 | 0.5651 | 0.1148 | 0.014 |
| Wild-Type | *elt-1(ku491);*  *daf-12(rh61rh411)* | 0.3583 | 0.0052 | 0.0061 | 0.0007 | 0.0359 |
| *daf-12(rh61rh411)* | *elt-1(ku491)* | 0.0617 | > 0.9999 | 0.9063 | > 0.9999 | 0.9905 |
| *daf-12(rh61rh411)* | *elt-1(ku491);*  *daf-12(rh61rh411)* | 0.0237 | 0.3901 | 0.9413 | 0.6017 | 0.9999 |
| *elt-1(ku491)* | *elt-1(ku491);*  *daf-12(rh61rh411)* | 0.9996 | 0.4575 | 0.328 | 0.4575 | 0.9997 |
