# Supplementary material for: The GATA Factor elt-1 Regulates C. elegans Developmental Timing by Promoting Expression of the let-7 Family MicroRNAs
Source: PLoS Genet. 2015 Mar 27;11(3):e1005099. doi: 10.1371/journal.pgen.1005099 (PMC4376641; doi:10.1371/journal.pgen.1005099)
Supplement: S1 Text — (DOCX) [file pgen.1005099.s007.docx]

**Supplementary Information**

**Supplemental Materials and Methods**

**Screening and mapping**

A strain with the genotype *dpy-20(e1282)* IV; *wIs51* V; *daf-12(rh61rh411)* X was constructed and screened for F2-progeny with increased seam cells following EMS-mutagenesis [47]. *ku491* was one of more than 10 alleles isolated after screening about 30,000 genomes. Seven of these alleles were initially mapped to chromosome regions by standard SNP mapping after generating Hawaii/Bristol hybrid strains [55]. For this study, *ku491* was further mapped to a region between *dpy-20* and *unc-24* and further fine 3-point mapping was performed as previously described [56,57] using variant identified by whole-genome sequencing. Sequencing was performed using an Illumina HiSeq 2000 following library construction with an Illumina DNA sequencing sample preparation kit (Illumina Inc.; San Diego, CA). Raw data was analyzed with FastQ, reads were mapped to the *C. elegans* genome with both Bowtie2 and BWA software packages, post-alignment variant-calling was performed with SAM tools, and the results were visualized with Integrated Genome Viewer (Broad Institute; Cambridge, MA) in the region 3-point mapping suggested.

**Assessment of Phenotypes**

The phenotype of strains was scored as follows. For all strains scored for seam-cell number, the seam-cell-specific nuclear-localized GFP reporter *wIs51* was crossed into the strain and scored at 64x magnification using GFP optics on a Zeiss AxioPlan 2 Microscope configured for Differential Interference Contrast (DIC) imaging with epifluorescence capability. Strains were scored during the period between completion of the previous molt and the initiation of the following round of seam-cell mitosis [33]. The absence or presence of alae was scored by Nomarski optics at 64x magnification. The ajm::gfp adherens junction GFP marker *kuIs47* was examined at 100x magnification. To determine the L4 bursting-vulva rate, fed L1 animals were picked to fresh plates and allowed to develop to L4 and adulthood, and the number of animals picked at L1 and with the described L4 phenotypes was recorded. The L4 bursting vulva rate was calculated as the number of animals with a burst vulva during the L4 period divided by the sum of that number and the number of animals that survived to past the L4 molt without a burst vulva. Feeding RNAi was performed as described [48], starting at the L1 stage. Statistical comparisons of seam-cell phenotypes were performed with Prism 6 as one-way ANOVAs with p-values for each comparison calculated with Bonferroni’s multiple comparisons method. For L4 bursting vulva rate, data was analyzed with Prism 6 and p-values were calculated by 2-tailed binomial t-test. Seam cell data is shown as a scatter plot with the mean.

**Alignment and structural analysis of ELT-1 Zn-finger DNA binding domains**

A multiple alignment was performed of the ELT-1 protein sequence with the protein sequences for Drosophila *grail*, *D. rerio* GATA3, *M. musculus* GATA1, and *H. sapiens* GATA1 using ClustalX. The PDB structure 3VD6 was visualized with SwissPDB Viewer 4.1.0. 3VD6 is the structure of the Zn-finger DNA binding domains of *M. musculus* GATA1 crystalized with palindromic DNA [39].

**Quantitative measurement of mRNA and miRNA expression levels**

RT-qPCR was performed to measure abundance of the *lin-28, hbl-1*, and *lin-41* mRNAs. Stage-specific samples of the indicated genotypes were collected from mixed-stage plates based on gross appearance and vulval morphology. Total RNA was purified with TRIZOL and cDNA was generated with SuperScript Reverse Transcriptase III (Invitrogen). qPCR was performed using a Rotor-Gene 3000 instrument. Relative gene expression was calculated as previously described [50]; *eef-2* was used as a loading control and targets dCts were normalized to the L1 arithmetic mean dCt for each gene. The qPCR primers had the following sequences: TAAACCATACTACCACCTACCT and AACAGGTGCAATCAGTTCTAT for *lin-28*, CTCGTCTAGTGACCCATTCT and ACGCCCGAACATTGATAAG for *hbl-1*, TGGCTCATTTGGACTTGGAC and GGACCACCGAGAGACGAATA for *lin-41*, and TCTTCTCGAGCCAGTCTACTT and AACGTGTCCTCTTCTTCTGTTC for *eef-2*. Expression of miRNAs was measured using TaqMan MiRNA assay kits (Invitrogen Corp.) according to the manufacturer’s instructions. 5ng of total RNA, prepared as above, was used as input into all reverse-transcription reactions. Data was analyzed by the ddCt method with normalization to the snoRNA U18 as recommended by the manufacturer. The fold-change value of each data point was used for graphing and statistical analysis with Prism 6 software (GraphPad); statistical analysis was performed using 2-way ANOVA with p-values calculated using Sidak’s multiple comparisons test.

***Expression of an* in vivo *reporter of regulation on the* lin-41 *3’ Untranslated Region***

The *pkIs2084* integrated reporter for the *lin-41* 3’ Untranslated Region (UTR) [44,3] was obtained and crossed into the indicated strains. Staining for lacZ activity was performed as described [51] and strains of the indicated genotypes/stages were scored for expression; saturated staining at any point in the animal was scored as strong positive, visible but unsaturated staining as weak positive, and undetectable staining as negative. All four strains were prepared in parallel with minimal gaps in processing times between strains. The stain was allowed to develop for approximately 6 hours. Data was analyzed with Prism 6 and p-values calculated with Fischer’s exact test.

**Supplemental References**

55. Davis MW, Hammarlund M, Harrach T, Hullett P, Olsen S, et al. (2005) Rapid single nucleotide polymorphism mapping in C. elegans. BMC Genomics 6: 118. doi:10.1186/1471-2164-6-118.

56. Fay D (2006) Genetic mapping and manipulation: chapter 3--Three-point mapping with genetic markers. WormBook: 1–7. doi:10.1895/wormbook.1.92.1.

57. Fay D (2006) Genetic mapping and manipulation: chapter 5--SNPs: three-point mapping. WormBook: 1–4. doi:10.1895/wormbook.1.94.1.
